# Home Physical Exercise Protocol for Older Adults, Applied Remotely During the COVID-19 Pandemic: Protocol for Randomized and Controlled Trial

**DOI:** 10.3389/fpsyg.2022.828495

**Published:** 2022-02-04

**Authors:** Anderson D’Oliveira, Loiane Cristina De Souza, Elisa Langiano, Lavinia Falese, Pierluigi Diotaiuti, Guilherme Torres Vilarino, Alexandro Andrade

**Affiliations:** ^1^Health and Sports Science Center – CEFID/Santa Catarina State University, UDESC, Florianópolis, Brazil; ^2^Laboratory of Sports and Exercise Psychology – LAPE, Florianópolis, Brazil; ^3^Department of Human Sciences, Society and Health/University of Cassino and Southern Lazio, Cassino, Italy

**Keywords:** exercise training, e-health, physical activity, aged, telemedicine, mental health

## Abstract

The emergence of the new coronavirus (COVID-19) at the beginning of 2020, considered a public health emergency due to its high transmission rate and lack of specific treatment, led many countries to adhere to social isolation. Although necessary, social isolation causes important psychological changes, negatively affecting the health of the population, including the older population. The aim of this study is to propose a 4-week, home-based physical exercise protocol for older people in social isolation and evaluate whether will promote positive changes in psychological variables such as anxiety, mood, depression, and stress, and in the variables sleep, quality of life, and physical capacities in the older adults. The sample will be selected in a probabilistic way from individuals aged 60 years or more from the city of Itajaí (Santa Catarina, Brazil). Of these, half will perform a home-based resistance training protocol, with 3 weekly sessions, for 4 consecutive weeks. For group allocation, patients will be randomized with a computer-generated 1:1 allocation to the physical exercise (PE) group or control group. Outcomes will be depressive symptoms, sleep quality, quality of life, stress, mood states, anxiety, and functional capacity, evaluated at baseline, after 4 weeks, and after 15 days of follow-up. This study will offer a home-based exercise protocol for older adults, with load progression and remote monitoring, thus filling a gap in the provision of PE in this population. The results will be able to identify possible improvements not only in physical health, but also in quality of life and mental health.

**Clinical Trial Registration:** The trial registration was carried out in the Brazil Clinical Trials Registry (RBR-5qh6f3v). (https://ensaiosclinicos.gov.br/rg/RBR-5qh6f3v).

## Introduction

The emergence of the new coronavirus (COVID-19) caused a pandemic considered a public health emergency by the World Health Organization (WHO). Due to the high transmission and lack of specific treatment, many countries have adopted restrictive measures, recommending social isolation and quarantine periods to contain the spread of the virus (Kraemer et al., 2020; World Health Organization [WHO], 2020a). Even today, there are countries that continue to recommend social isolation and prohibit agglomerations to avoid overloading health systems (Armitage and Nellums, 2020; Ferguson et al., 2020; Camuñas, 2021; Diotaiuti et al., 2021), this new situation implies challenges for different populations, such as athletes, workers and even the elderly (Andreato et al., 2020; Hernández-Hernández and Sancho-Gil, 2021).

Although necessary, social isolation causes important psychological changes, negatively affecting the health of the population, including the older population (Kenyon, 2020; Kraemer et al., 2020). With advancing age, a reduction in social interactions is common due to retirement and health conditions (Gao et al., 2018; Segel-Karpas et al., 2018), and this has been further aggravated during the COVID-19 pandemic. Studies indicate that being socially connected is related to positive outcomes of physical and psychological health and longevity in older adults (Gao et al., 2018; Segel-Karpas et al., 2018). More specifically, isolation is a risk factor for depression, anxiety disorders, stress, feelings of confusion, weakness, loneliness, anger, suicide, and worsening of existing psychiatric symptoms (Brooks et al., 2020; Sepúlveda-Loyola et al., 2020; Vindegaard and Benros, 2020; World Health Organization [WHO], 2020b).

In addition, social isolation facilitates a predominantly sedentary lifestyle, which is also associated with negative health effects (Reed et al., 2011; De Rezende et al., 2014; World Health Organization [WHO], 2020b; Reyna et al., 2021), implying a physical deconditioning, not only related to musculoskeletal levels, but the resulting negative metabolic changes (Montero et al., 2017; Bowden Davies et al., 2019), being related to greater chances of mortality, metabolic diseases, and cancer, among others (De Rezende et al., 2014; Gilchrist et al., 2020; Park et al., 2020). Therefore, strategies to reduce sedentary behavior in older adults are essential to improve quality of life, physical and mental health (Sepúlveda-Loyola et al., 2020; da Cruz et al., 2021) and physical deconditioning (Santy-Tomlinson, 2021).

Physical exercise (PE) is indicated to combat sedentary lifestyle, as well as being used as a means of prevention and treatment of several common diseases in aging, such as heart disease, diabetes, osteoporosis, hypertension, cancer, fibromyalgia, anxiety disorders, and depression (Debussche et al., 2012; Reif et al., 2013; Andrade et al., 2017a,b; Sties et al., 2018; da Silva et al., 2020). In addition, PE helps to promote health, providing an increase in quality of life (Haraldstad et al., 2017) and physical and psychological well-being (Andrade et al., 2018; Vilarino et al., 2021), and positively influencing affective and social relationships at all ages (Camuñas, 2021; Ni et al., 2016; Callow et al., 2020).

The need to keep the population physically active during isolation and social distancing has led to organizations such as the American College of Sports Medicine [ACSM] (2020), American Heart Association [AHA] (2020), American Physical Therapy Association [APTA] (2020), International Association of Physical Therapists working with Older People [IPTOP] (2021), and World Health Organization/EUROPE [WHO/EUROPE] (2020) to recommend the practice of PE remotely, such as online videos, applications, and online platforms for mobile phones and tablets through systems connected to the internet (Sepúlveda-Loyola et al., 2020). However, as far as we know, only one study was published verifying the effect of remote PE practice in older adults (Vitale et al., 2020). The authors showed positive results, indicating the possibility of performing PE remotely in this population, however, the topic needs further investigation.

Among the possibilities of PE practice during the period of social isolation, resistance training (RT) stands out, as it does not require equipment and can be performed without leaving the house and in small spaces. RT has numerous benefits for the older population, such as increased muscle strength, quality of life, and psychological well-being (Andrade et al., 2020; Vitale et al., 2020; da Cruz et al., 2021; Di Lorito et al., 2021). However, prescription and monitoring of RT remotely require different strategies, which should allow the same benefits as when monitored in person.

Thus, there is a need for studies that specify in detail the intervention used to provide guidelines for the practice of professionals. Furthermore, these studies should be controlled and randomized to reduce the risk of bias and increase safety regarding the expected effects on physical and psychological variables for the older population (Kenyon, 2020; Kraemer et al., 2020; Yamada et al., 2021). It was identified that there is no established protocol that indicates the best intensity, volume, and load progression and that presents parameters for monitoring and evaluation. Therefore, the creation and use of PE protocols for older adults through online technologies and with guidance from trained health professionals could represent an alternative to improve the physical and mental health of older adults during a period of isolation and social distancing, reducing psychological and affective disorders, which have been associated with increased morbidity caused by COVID-19 (Armitage and Nellums, 2020; Santini et al., 2020).

The hypothesis is that the remote PE protocol will promote changes in the psychological outcomes of depression, anxiety, mood states, and stress, and on the outcomes of sleep quality, quality of life, and physical abilities of older people in social isolation. In this sense, the aim of this study is to propose a 4-week (3x/week) home-based physical exercise protocol applied remotely to older people in social isolation.

## Materials and Methods

### Study Design

**FIGURE 1:**
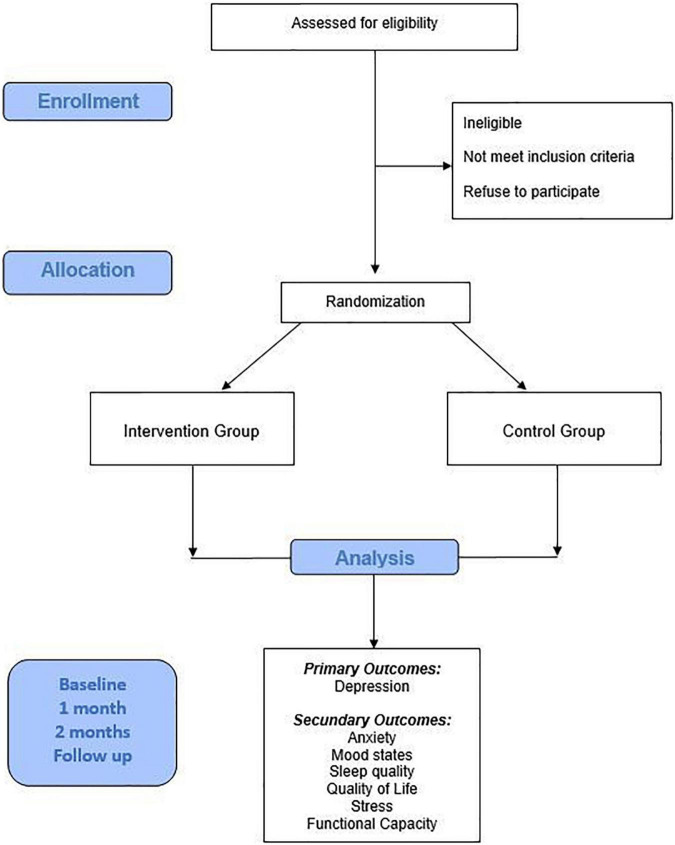
Flowchart demonstrating the participant selection process and the steps of the study protocol.

This is a protocol study for a randomized controlled trial (RCT), parallel-group, developed according to Standard Protocol Items: Recommendations for Intervention Trials (SPIRIT) (Chan et al., 2013) (Figure 1). This protocol was developed for a 4-week PE intervention and monitoring of a control group.

### Participants

The sample will be selected probabilistically with individuals aged 60 years and over from the city of Itajaí (Santa Catarina, Brazil). They will be recruited from a telephone directory of people registered with the Municipal Secretariat for the Promotion of Citizenship. Participants who meet the inclusion criteria will be randomized equally between two groups; the experimental group who will carry out the PE protocol (PEP) and the control group (CG) who will be instructed not to perform any type of PE and to follow their usual daily routines. For group allocation, patients will be randomized with a 1:1 computer-generated allocation^1^ and the researcher responsible for the randomization will not be involved in the evaluations.

To participate in the study, the following inclusion criteria will be respected: individuals of both sexes, aged ≥ 60 years (no maximum age limit); with internet access; able to understand and use the smartphone; with the physical and cognitive ability to understand and perform the exercises safely. The exclusion criteria will consist of elderly people who performed physical activity and/or exercise in the last 3 months, according to the criteria recommended by the World Health Organization (World Health Organization [WHO], 2011) (walks of 150 min per week and/or resistance training with weight for 75–150 min) and elderly people with any condition physical or cognitive that makes it impossible to practice the proposed exercises at home.

### Outcome Measures

The evaluations will be carried out before the beginning of the intervention, after 4 weeks of the intervention, and 15 days after the end of the intervention. In the initial assessment, a characterization questionnaire will be applied to obtain socioeconomic and health information.

The primary outcome will be the variable depression (Gorenstein and Andrade, 1996) and the secondary outcomes will be mood (Rohlfs et al., 2008), stress (Luft et al., 2007), sleep quality (Buysse et al., 1989; Bertolazi et al., 2009), quality of life (Power et al., 2005), anxiety (Beck et al., 1988) and physical abilities (Hlatky et al., 1989) of the older adults. The analyzed variables and the respective instruments are listed below in Table 1.

**TABLE 1:** List of variables, instruments used, interpretation and reference.

Variables	Instruments	Interpretation of instruments/cutoff points	Authors (year)/articles
Depressive symptoms	BDI	These ups and downs are considered normal (1–10)/Mild mood disturbance (11–16)/Borderline clinical depression (17–20)/Moderate depression (21–30)/Severe depression (31–40)/Extreme depression (Over 40)	Gorenstein and Andrade (1996)/Validation of a Portuguese version of the BDI and State-Trait anxiety inventory in Brazilian subjects
Sleep quality	PSQI	Good sleep (0–4)/Sleep Disorder (5 or more)	Buysse et al. (1989)/The PSQI: a new instrument for psychiatric practice and research.
	Ese-Br	Scores above 10 suggest the diagnosis of excessive daytime sleepiness	Bertolazi et al. (2009)/Portuguese-language version of the Epworth sleepiness scale: validation for use in Brazil
Quality of life	WHOQOL—OLD	Need to improve (1–2.9)/Regular (3–3.9)/Good (4–4.9)/Very good (5)	Power et al. (2005). Development of the WHOQOL-old module
Stress	PSS	Scores can range from 0 to 56/Not the cut-off point.	Luft et al. (2007). Brazilian version of the Perceived Stress Scale: Translation and validation for the elderly
Mood states	BRUMS	Score can vary from 0 to 16/Not the cut-off point.	Rohlfs et al. (2008). BRUMS: an instrument for early detection of overtraining syndrome.
Anxiety	BAI	Scores between (0–21) indicates very low anxiety/(22–35) indicates moderate anxiety/A grand sum that exceeds 36 is a potential cause for concern.	Beck et al. (1988). An inventory for measuring clinical anxiety: Psychometric properties.
Functional capacity	DASI	The final score varies between zero and 58.2 points/Not the cut-off point.	Hlatky et al. (1989). A brief self-administered questionnaire to determine functional capacity (the Duke Activity Status Index).

*BAI, Beck Anxiety Inventory; BDI, Beck Depression Inventory; BRUMS, Brunel Mood Scale; DASI, Duke Activity Status Index; Ese-Br, Epworth Sleepiness Scale; PSQI, Sleep quality index (Pittsburgh); PSS, Perceived Stress Scale; WHOQOL—OLD, World Health Organization Quality of Life Group.*

### Adverse Events

Any adverse effects observed or reported by patients will be recorded and considered in the study results. In addition, these patients will be referred to medical care for proper treatment.

### Data Collection and Procedures

**FIGURE 2:**
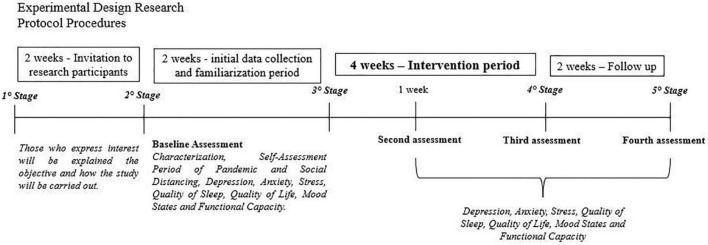
Data collection procedures and steps.

The first contact will be made *via* phone call by the research team, for those who declare an interest, the objective of the study and how it will be conducted will be explained (need to use a cell phone, data collection through an online form, intervention through videos and guidance), as well as the importance of adhering to the program. After verbal agreement to participate, a link will be sent by phone or messaging app, to sign the free and informed consent form and complete the baseline evaluation form, along with an explanatory video with information for possible doubts. Participants will perform the baseline assessment within a 2-week period before the start of the intervention. Support will be offered to the participants, to minimize the limitation of poor technology skills. In Figure 2 it is possible to observe the steps of the protocol.

The researchers responsible for delivering and assisting with the assessments will not know which group the individual is randomized to, that is, different researchers will have to send the informative videos and collect the weekly information about the exercises.

### Experimental Group

The PE will be carried out by the participants in a home-based format and all contact will be made by phone or messaging app. After the baseline assessment, the intervention will start and after 4 weeks of training, a post-intervention evaluation will be carried out, following the same procedure: link to the online form, accompanied by an explanatory video. Fifteen days after completion of the PE program, a follow-up assessment will be carried out. All participants will be able to contact the researchers in case of doubts regarding data collection and intervention. The importance of maintaining their routine medication use will be highlighted.

#### Intervention: Physical Exercise Protocol

The PE protocol was specifically developed to be performed by older individuals remotely in their homes, under the supervision of physical education professionals and physiotherapists. Physical Education Professionals and Physiotherapists are researchers experienced with prescribing and supervising RT for the elderly population. Supervision will be performed through explanatory videos, text messages, and, when necessary, by phone call. Professionals will be available for possible queries, *via* messaging application and call or e-mail.

Once a week, videos will be sent to the intervention group in the messaging application. The videos will contain exercises demonstrated by a professional and detailed explanations through audio and subtitles. To reinforce the performance of the PE, two messages will be sent per week to reinforce the practice instructions.

The dosage of the intervention will consist of 3 weekly sessions lasting 40–70 min each, with a 1-day break between them, for 4 weeks, totaling 12 sessions. The 4-week intervention will be divided into three periods to facilitate exercise progression; the first week (period 1) will be for adaptation and familiarization, and the second week (period 2), and third and fourth weeks (period 3) for the progression of repetitions and sets, as explained in Table 2 (Bird et al., 2005). The sessions will consist of exercises with free weights, strengthening exercises, and stretching. By performing at least 50–60 min in three sessions per week, older adults can reach the level of physical activity recommended by the WHO (World Health Organization [WHO], 2011) of 150 min per week.

**TABLE 2:** Home physical exercise protocol for older adults during the COVID-19 pandemic.

Intervention weeks	Weekly frequency	Intensity	Volume*	Number of series	Speed in concentric and eccentric phase	Duration of each session
1°Week	3x	10 Repetitions	90	1	≈3 Segundos	36 min
2°Week	3x	10 Repetitions	180	2	≈3 Segundos	51 min
3°Week	3x	15 Repetitions	270	2	≈3 Segundos	1 h–1:06 min
4°Week	3x	15 Repetitions	270	2	≈3 Segundos	1 h–1:06 min

*The volume was calculated by multiplying the number of exercises, series and repetitions (Bird et al., 2005).

The session will start with a 10-min warm-up consisting of rotation exercises for the upper and lower limb joints. Next, 30–40 min of muscle strengthening exercises will be performed, where the load will be the body weight and 500 ml water bottles (equivalent to 500 g), ending with 10 min of stretching. There will be no rest intervals between exercises due to the low intensity and volume.

During the sessions, the main muscle groups involved in the exercises will be the pectoralis major, latissimus dorsi, biceps brachii, triceps brachii, deltoids, glutes, quadriceps, hamstrings, and calves. The following exercises will be performed: Sit and stand up from a chair, bench press, unilateral row, knee flexion and extension, elbow flexion, lateral raise, plantar flexion, and abdominal contraction. The older adults will only perform the exercises applied in the study. A detailed description of the exercise protocol for each week can be found in Supplementary File 1.

As this is an older population, there is a need to maintain effective communication to reduce risk and promote greater safety among participants. For this reason, participants will be instructed and encouraged to perform their movements within available ranges, respecting their limits, using a chair support, and remaining comfortable. Through the informative videos, it will be explained in a simple way how they might feel during the exercise and what is the acceptable level of tiredness. Weekly contact will also be made to track possible difficulties, doubts, and adverse effects, such as pain, discomfort, fatigue, or dizziness, for example. If there are adverse effects, corrections will be investigated and made, however, without prejudice to the objective of the exercise. In addition, through the initial assessment it will be possible to analyze the health profile of the participants, which makes it possible to advance corrections for each one, if necessary.

To access and evaluate adherence, during the weekly contact, mentioned previously, the researcher will ask to confirm that they have completed the proposed exercises and the corresponding performed date. To ensure that the proposed intervention was performed indeed, the participants must also keep a diary, reporting the number of sets of each exercise and the duration of each session, this information will be collected by the researcher who will contact the participants (Lambert et al., 2017; Vitale et al., 2020).

### Control Group

To participants allocated to the control group a link will be sent to sign the free and informed consent form and complete the baseline evaluation form, along with an explanation video with information on possible doubts. The participants will also be asked to maintain their normal activities (including taking medications) and not to perform PE. After a period of 4 weeks, a link with an online form and an explanatory video for evaluation will be sent again. Fifteen days after this evaluation, the follow-up form and video will be sent. After the research period, a new invitation to carry out the proposed physical exercise program will be extended to the participants of the voluntary control group.

### Analysis

#### Sample Size Calculation

The sample size was calculated using the GPower 3.1 program. Considering an alpha risk of 0.05 and power of 0.95, a sample with at least 56 participants is required, randomized into the two groups. We anticipate that 20% of the participants will drop out, so will recruit an additional 12 (6 in each group).

#### Statistical Analysis

Data analysis will be performed using IBM SPSS software (version 20.0). Descriptive statistics, with mean, standard deviation and percentage. For the inferential analyses, two-way ANOVA with repeated measures will be used and the Bonferroni *post hoc* comparison test will be utilized for a comparative analysis of the results of the control group with the experimental group and within-group changes between pre- and post-intervention periods. The α established as a level of significance will be *p* < 0.05 (95%) for all hypothesis tests performed.

### Ethics and Dissemination

The project was approved by the Ethics Committee for Research with Human Beings of the State University of Santa Catarina, under Certificate of Presentation of Ethical Appreciation (CAEE number) 40392220.2.0000.0118, approval number 4.617.650. The status of approval can be consulted on the website https://plataformabrasil.saude.gov.br. Written informed consent will be obtained from each respondent before data collection.

## Discussion

In view of the challenges that the COVID-19 pandemic has presented to the entire world population, one of the biggest obstacles debated in the literature is how to preserve physical and mental health even in this moment of social isolation (Rossi et al., 2020; Batsis et al., 2021). Thus, among the intervention strategies studied, PE represents a viable possibility (Vitale et al., 2020).

It is known that PE is an effective approach to improve the general health of older people. Two recently published systematic reviews with meta-analysis demonstrated that PE interventions increase muscle strength, dynamic and static balance, gait speed, and functional capacity, decrease the incidence of falls and levels of depression and anxiety, and improve mood. Although RT is one of the most effective modalities (Kazeminia et al., 2020; Di Lorito et al., 2021), these results are based on interventions carried out in person, with the participants being guided and supervised throughout the PE session, which is hardly viable in this period of pandemic. Thus, the prescription and supervision of PE need to be re-evaluated to ensure the safety and effectiveness of training in older adults.

Despite this, minimally supervised home-based RT has shown effective results for strength gains and health improvement in older adults, in addition to being a safe and cost-effective intervention (Kis et al., 2019). The home-based PE prescription strategy follows the WHO guidelines, which recommend weekly sessions of 150–300 min, with moderate intensity (Bull et al., 2020), however, these guidelines are general and do not have practical guidelines that can be used immediately for exercise prescription.

Since the beginning of the pandemic, to our knowledge, only one randomized clinical trial has been carried out with home-based PE for older adults. In that study, participants performed 6 months of RT, totaling 96 sessions. The results indicated significant improvements in the intervention group in the outcomes related to muscle strength and physical performance, however, the sample consisted of only nine individuals, which limits the results (Vitale et al., 2020). Thus, some issues related to prescription need to be further investigated when considering home-based interventions to assess both the physical and mental health of older adults.

This gap in the literature can be filled with a systematic and specific home-based PE protocol through an RCT, as a form of non-pharmacological treatment to improve the physical and mental health of older adults, considering that PE practices are already established and considered beneficial on a large scale in international scientific publications (Lautenschlager et al., 2004). However, it is necessary to understand these data in a current scenario, with a perspective on the physical and mental health of older adults, through a PEP with safe remote monitoring. For this, the PEP was developed to meet current demands, promoting benefits in depression, anxiety, mood, sleep quality, stress, quality of life, and functional capacity.

The creation of this PE protocol through remote monitoring by health professionals will be an important ally to help prescribe PE for older adults in the midst of the pandemic caused by COVID-19. However, it has some limitations, including the difficulty of using and accessing technologies by the elderly population, which limits this intervention to those who have access and know how to use cell phones with internet. As there is no interaction at the time of exercise practice, attendance depends on the participant reports, considered a bias in the study. Another limitation, as this is an active intervention, it is not possible to blind the participants, which can also generate bias.

This protocol will serve as an excellent strategy for new public policies in several countries. Older people are a growing demographic group worldwide, but the increase in life expectancy must be accompanied by an equally good quality of life from these gained years. Physical activity certainly behaves as a protective factor at all ages, so this protocol could be very important to reach many elderly people who for various reasons cannot go to the gym but would be willing to follow this type of training at home. It is a unique PE protocol, developed in a systematic way, that is simple to perform, and requires little equipment and financial resources, thus contributing to its implementation and cost-effectiveness, and making it accessible to everyone.

## Ethics Statement

The studies involving human participants were reviewed and approved by the Ethics Committee for Research with Human Beings of the State University of Santa Catarina, under protocol 40392220.2.0000.0118. The patients/participants provided their written informed consent to participate in this study.

## Author Contributions

AD: conceptualization and writing. LD: writing. GV: writing and review. EL, LF, and PD: review. AA: writing—review and supervision. All authors have contributed significantly to this manuscript and agreed with its content.

## Conflict of Interest

The authors declare that the research was conducted in the absence of any commercial or financial relationships that could be construed as a potential conflict of interest.

## Publisher’s Note

All claims expressed in this article are solely those of the authors and do not necessarily represent those of their affiliated organizations, or those of the publisher, the editors and the reviewers. Any product that may be evaluated in this article, or claim that may be made by its manufacturer, is not guaranteed or endorsed by the publisher.

## Footnotes

1
http://www.randomization.com
